# Total and Methylmercury in Soft Tissues of White-Tailed Eagle (*Haliaeetus albicilla*) and Osprey (*Pandion haliaetus*) Collected in Poland

**DOI:** 10.1007/s13280-014-0533-8

**Published:** 2014-05-29

**Authors:** Elzbieta Kalisinska, Jerzy Gorecki, Natalia Lanocha, Anna Okonska, Javier B. Melgarejo, Halina Budis, Izabella Rzad, Jerzy Golas

**Affiliations:** 1 Department of Biology and Medical Parasitology, Pomeranian Medical University, Powstancow Wielkopolskich Av. 72, 70-111 Szczecin, Poland; 2 Department of Coal Chemistry and Environmental Sciences, Faculty of Energy and Fuels, AGH University of Science and Technology, Mickiewicza Av. 30, 30-059 Kraków, Poland; 3 Department of Health Education, University of Szczecin, al. Piastow 40b bl. 6, 71-065 Szczecin, Poland; 4 Department of Ecology and Environment Protection, University of Szczecin, Waska 13, 75-415 Szczecin, Poland

**Keywords:** Falconiformes, Piscivorous birds, Organic and inorganic mercury, Accumulation, Tissue differences

## Abstract

Mercury (Hg) contamination in piscivorous birds, especially methylmercury (MeHg), has been drawing much attention worldwide in regard to its bioaccumulation and biomagnification in food chains. In this study on Hg in the soft tissues of white-tailed eagles (*n* = 22) and ospreys (*n* = 2) from Poland, total Hg (THg) range was 0.15–47.6 while MeHg range was 0.11–8.05 mg kg^−1^ dry weight. In both species, median THg and MeHg concentrations were lower in the muscle and brain than in the liver and kidney. Median nephric residues were just under 3 and 5 mgTHg kg^−1^ or 0.9 and 3.7 mgMeHg kg^−1^ for white-tailed eagle and osprey, respectively. In Norwegian data from the 1970s and in our results, MeHg in the muscle of white-tailed eagle was ~60 % THg (%MeHg = MeHg/THg × 100), lower than in other piscivorous birds. A clear similarity in THg tissue levels was found between Polish and German populations of white-tailed eagles.

## Introduction

According to the European Union Bird Directive (2009/147/EC), the white-tailed eagle *Haliaeetus albicilla* and osprey *Pandion haliaetus* are rare protected species of Falconiformes. In Europe, breeding populations of white-tailed eagle and osprey are estimated at 5000–6600 and 7600–11 000 pairs, respectively. The white-tailed eagle has its strongholds in Norway and Russia, with significant populations in Sweden, Poland and Germany, with key breeding areas of the osprey in Sweden, Russia and Finland (BirdLife International 2004). In Europe, the migratory status of the white-tailed eagle is ‘resident’ or ‘mainly resident’ yet the osprey is full migrant. Both species are long lived and in the wild can live up to 25 years. The eagle matures sexually at 5 years and osprey at 3 years. Both are top predators and their diet is mainly fish, although the white-tailed eagle feeds on waterfowl and animal carcasses (Forsman 1999).

As piscivorous species, the white-tailed eagle and osprey are exposed to chronic or acute poisoning with mercury (Hg), especially methylmercury (MeHg), in their food (Holt et al. 1979). Research from the 1950s and later showed that among the various Hg species present in nature, MeHg is the main cause of neurological, neuromotorical, behavioural and reproductive disorders in warm-blooded vertebrates (Clarkson and Magos 2006; Rutkiewicz et al. 2011). The most considerable Hg poisonings of birds of prey were recorded between 1960 and 1980 when agriculture in developed countries based on the use of pesticides (especially, fungicides containing MeHg) for grain seed dressing. The treated grain was consumed by small birds and mammals, which in turn were a source of food for terrestrial predators. Also the fungicides washed from fields reached water bodies and contaminated aquatic food webs, leading to a catastrophic decrease of local populations of fish-eating birds (white-tailed eagle, bald eagle *Haliaeetus leucocephalus* and osprey) in Europe and North America. Moreover, MeHg undergoes biomagnification, especially in aquatic ecosystems (Scheuhammer et al. 2007). Despite the abandonment of Hg-pesticides in Europe and North America in 1980–1990s and many attempts to decrease anthropogenic Hg emissions, its various forms are still present in the environment worldwide (Larson 2014).

Birds are mainly exposed to Hg coming from the alimentary tract. Their food contains inorganic Hg (InHg) and organic forms, among which MeHg is the most prevalent. The absorption of MeHg and InHg from the avian digestive tract may be 99 and 20 %, respectively (Gochfeld 2003; Clarkson and Magos 2006). The greatest amounts of MeHg can be found bound in the plumage (70–90 % of total Hg, THg, accumulated in the avian body). Importantly, this MeHg is only transported to growing feathers to be shed during moulting. Half of the remaining THg is accumulated in muscles and the other half in other tissues, especially in the liver and kidneys (DesGranges et al. 1998). At the same time, they are the most efficient at MeHg demethylation, which results in the production of InHg and dynamically changing proportions of MeHg and InHg. This process occurs in the brain too. Muscles, feathers and eggs show little or no evidence of the demethylation (Scheuhammer et al. 2008). There are less data on the accumulation of THg and MeHg in the muscle and brain in piscivorous birds than the liver and kidneys (Shore et al. 2011). Muscles, unlike the liver and kidneys, are the main consumable part of the bird body and largely contribute to the transfer of MeHg to predators and scavengers. It is, therefore, justified to study not only hepatic and nephric THg and MeHg concentrations, but also their remains in avian muscle and brain (as a target organ of MeHg).

In formulating our hypothesis and the aim of ecotoxicological research, we took into account: special interest of European Union (EU) in the protection and monitoring of rare species; dynamic changes in the environment in Europe induced by socio-economic transformations; and potential exposure piscivorous birds to Hg. Our hypothesis assumed that the abandonment of Hg-pesticides has contributed to a decreased exposure of fish-eating Falconiformes. The verification of the hypothesis was conducted in the investigation of THg and MeHg residues in soft tissues of two rare piscivorous species: the white-tailed eagle and the osprey, collected in Poland after 1995, and comparison of the data with the analogous data published so far. In Europe, the current study is the second to investigate both THg and MeHg levels in soft tissues of the aforementioned species, the first one was published in Norway in 1970s. Reports on THg in birds are more frequent and can be used for interregional comparisons.

## Materials and Methods

### Study Area

The current population of the white-tailed eagle in Poland is estimated at 670 breeding pairs. At the end of the 1990s, the greatest numbers of pairs were observed in north-western Poland (Voivodeship Zachodniopomorskie, VZach: *n* = 180) and north-eastern Poland (Voivodeship Warminsko-Mazurskie, VWar-Maz: *n* = 150) (Cenian et al. 2006). In central Poland, few nests are found mainly due to its more industrial and agricultural character, fewer water bodies and forested areas. In the Voivodeship Lodzkie (VLodz), the number of breeding pairs is ~15 (Anderwald et al. 2007). Many white-tailed eagles breeding in Poland winter in their own nests or move further west. The greatest wintering populations can be found in NW Poland, sometimes up to 200 individuals have been observed around the Szczecin Lagoon (Hauff and Mizera 2006).

Currently, about 25–30 pairs of osprey breed in Poland, mainly in northern and western part of the country (Mizera 2009). Throughout the country numerous ospreys migrate that breed in North Europe (Forsman 1999).

The Polish energy sector is based on coal combustion, which results in high Hg emission. However, a distinct 60 % decline in Hg emissions has been observed in Poland since the 1990s, down to 13.5 tonnes in 2010 (Panasiuk and Glodek 2013). In VZach, VWar-Maz and VLodz, Hg emissions in 2007 were, respectively, 0.51, 0.14 and 1.97 tonnes (Debski et al. 2009). A decrease in Hg concentrations was observed in fish from the Southern Baltic, potential food for osprey and white-tailed eagle. The fish examined between 1990 and 2010 had average concentrations <0.50 mgHg kg^−1^ wet weight (Polak-Juszczak 2013).

### Sampling

**Fig. 1:**
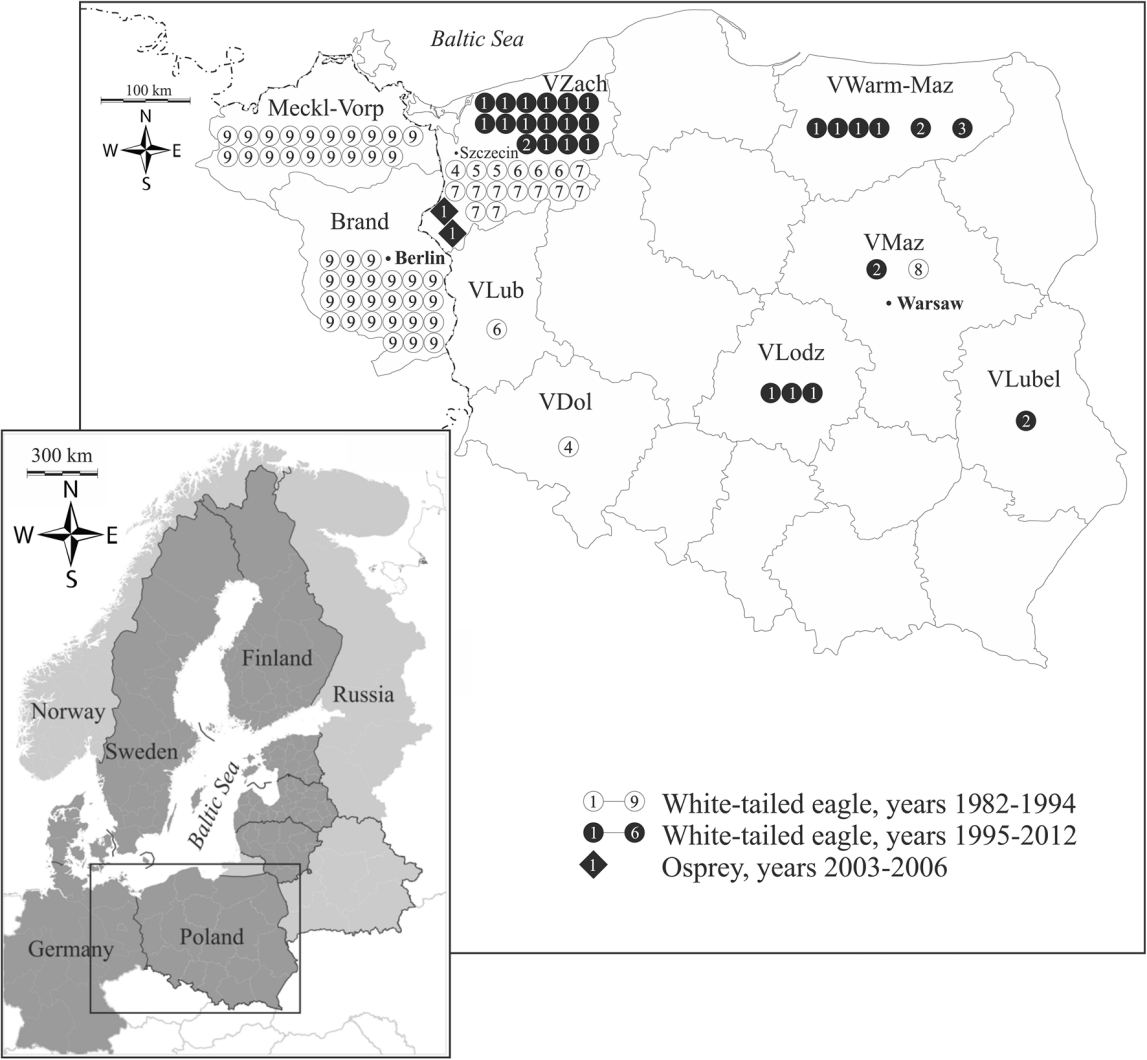
Location and number (expressed by number of symbols) of the white-tailed eagle and osprey in which soft tissues mercury concentration was studied in Poland (*VZach* Voivodeship Zachodniopomorskie, *VWar-Maz* V. Warminsko-Mazurskie, *VLub* V. Lubuskie, *VDol* V. Dolnoslaskie, *VLodz* V. Lodzkie, *VMaz* V. Mazowieckie, *VLubel* V. Lubelskie) and North-East Germany (*Meckl-Vorp* Mecklenburg-Vorpommern, *Brand* Brandenburg) in years 1982–2012. Sources of data: *1*. this study (black diamond and circles marked “1”); *2*. Komosa et al. (2009); *3*. Kitowski et al. (2012); *4*. Falandysz and Jakuczun (1986); *5*. Falandysz et al. (1987); *6*. Falandysz et al. (1988); *7*. Falandysz et al. (2001); *8*. Dittmann et al. (1990); *9*. Kenntner et al. (2001)

Between 1996 and 2012 twenty-two dead specimens of the white-tailed eagle were collected in three regions of Poland: VZach, VWar-Maz and VLodz. Two dead ospreys were found in VZach (Table 1; Fig. 1). The collection of carcasses of the species was performed in compliance with Polish law. Until analysis, the carcasses were stored at −20 °C. After defrosting, the specimens were classified to an age group (based on the morphological characteristics of the plumage and beak), and sex of birds was established by inspection of the gonads during autopsy (Forsman 1999). The 22 white-tailed eagles were divided into three age groups (immature, between 5 months and 4 years; subadult, 4th year; adult, 5 years and older) and the two ospreys into two age categories (immature and adult). Samples of liver, kidney and breast muscle were taken from all individuals. In 8 of the eagles, it was impossible to collect whole brains so only THg was determined (Table 1).

**Table 1:** Origin of soft tissues of the white-tailed eagle *Haliaeetus albicilla* and osprey *Pandion haliaetus* found in Poland between 1996 and 2012 (*F* female, *M* male, *im* immature, *subad* subadult, *ad* adult; voivodeships: *VZach* Zachodniopomorskie, *VLub* Lubuskie, *VWar-Maz* Warminsko-Mazurskie, *VLodz* Lodzkie)

Eagle ID	Sex and age	Location (voivodeship, district)	Collection	Cause of death and additional information	Assayed THg and MeHg (+) or THg only (±)			
					Liver	Kidney	Muscle	Brain
*Haliaeetus albicilla* (*n* = 22)								
HA-1	F, im	VZach, Goleniow	Jan 1996	Unknown	+	+	+	+
HA-2	M, im	VZach, Police	Aug 2005	Trauma (fractured wing)	+	+	+	+
HA-3	F, im 18 ms	VZach, Szczecin	Sept 2005	Train collision; ringed in E Germany	+	+	+	±
HA-4	F, im 10 ms	VZach, Gryfino	Feb 2010	Electric fan collision, emaciated; ringed in E Germany, Brandenburg	+	+	+	+
HA-5	M, im	VWar-Maz, Ilawa	Mar 2011	Disease	+	+	+	+
HA-6	M, subad	VZach, Bialogard	Feb 2009	Trauma (broken leg)	+	+	+	+
HA-7	F, subad	VZach, Goleniow	Aug 2009	Unknown	+	+	+	+
HA-8	F, subad, 5 ys	VZach, Gryfino	Mar 2010	Electric fan collision; ringed in E Germany, Brandenburg	+	+	+	+
HA-9	M, ad	VZach, Lobez	Feb 1996	Eagle attack	+	+	+	+
HA-10	M, ad	VZach, Gryfino	Aug 1996	Unknown	+	+	+	
HA-11	F, ad	VZach, Mysliborz	Nov 1998	Unknown	+	+	+	±
HA-12	F, ad	VZach, Goleniow	Feb 2007	Unknown	+	+	+	+
HA-14	F, ad	VLodz, Lowicz	Sep 2007	Shot gun	+	+	+	
HA-13	F, ad	VLodz, Laski	Dec 2007	Shot gun	+	+	+	
HA-15	M, ad	VLodz, Lowicz	Nov 2007	Unknown	+	+	+	
HA-16	F, ad, 16 ys	VZach, Goleniow	Nov 2008	Drowning, ringed in Poland	+	+	+	+
HA-17	M, ad	VZach, Gryfino	May 2008	Unknown (deformed beak)	+	+	+	+
HA-18	M, ad	VZach, Pyrzyce	Jan 2009	Unknown	+	+	+	
HA-19	F, ad	VZach, Kamien Pomorski	Mar 2009	Electric fan collision	+	+	+	
HA-20	M, ad	VWar-Maz, Ilawa	Mar 2011	Unknown	+	+	+	+
HA-21	F, ad	VWar-Maz, Ostroda	Mar 2011	Disease, emaciated	+	+	+	+
HA-22	M, ad	VWar-Maz, Ostroda	April 2012	Unknown (suspected poisoning)	+	+	+	+
*Pandion haliaetus* (*n* = 2)								
PH-1	F, im	VZach, Gryfice	Sep 2006	Shot gun	+	+	+	+
PH-2	F, ad	VZach, Gryfino	Sep 2003	Trauma	+	+	+	+

### Mercury Analysis

The assays involved determinations of tissue per cent water content (by weight) and concentrations of THg and MeHg. The avian samples were dried to constant weight (at 55°C) and then ground in the Planetary Mono Mill Pulverisette 6.

MeHg was determined in KOH extract using PDMS-GC-Pyr-AFS method described in details by Gorecki et al. (2013). The procedure was tested with the CRM DORM-2 (reference value of MeHg: 4.47 ± 0.32 mg kg^−1^). The obtained MeHg concentration in the DORM-2 was 4.38 ± 0.12 mg kg^−1^ (*n* = 4) and OC/RV = 97.99 % (where OC and RV are obtained concentration and reference value, respectively). THg concentrations in the samples (in KOH extracts) were determined using an Automated Mercury Analyzer MA-3000 (Nippon Corporation).

### Statistical Analysis

Statistical analyses were performed using StatSoft Statistica 9.1. Variation of the mean moisture among the avian tissues was evaluated by one-way ANOVA. Concentrations of THg and MeHg in the liver (L), kidney (K), muscle (M) and brain (B) were expressed in dry weight (dw). The percentage of MeHg in THg (%MeHg = MeHg/THg × 100) was also established. According to Norheim and Frøslie (1978), relative (to liver) concentrations of THg and MeHg were calculated as indices: THgK/THgL, THgM/THgL, THgB/THgL, MeHgK/MeHgL, MeHgM/MeHgL, MeHgB/MeHgL. The *Kolgomorov*-*Smirnov* test with Lilliefors correction showed that the distribution of THg and MeHg concentrations in the eagle samples deviated from the expected normal distribution, and so in the statistical analysis the comparison of the mean concentrations of Hg, non-parametric Kruskal–Wallis (K–W) or Mann–Whitney (M–W) tests were used when the number of means was respectively ≥3 or equal 2. Relationships between Hg concentrations among tissues and age group were evaluated by calculation of Spearman correlation coefficient (*r*_S_). The correlation coefficients and differences in mean moisture, absolute and relative Hg concentrations among tissues, and Hg concentrations between age, genders and locations were considered statistically with an alpha level of 0.05. Since osprey samples came only from two specimens, interspecific comparison was not performed.

## Results

The water content in the liver, kidney, muscle and brain of the white-tailed eagle was 71.3, 75.3, 70.1 and 80.7 %, respectively, with significant differences between the tissues (*F* = 48.5, df = 3, *p* < 0.001). The exception was the relation between L and M, with similar moisture. In the osprey tissues, the moisture was similar to that observed in the eagle.

### White-Tailed Eagle

Concentrations of THg and MeHg in the white-tailed eagle samples were within 0.15–47.6 and 0.11–8.05 mg kg^−1^, respectively (Table 2). The greatest variability was found for THg and MeHg concentrations in the kidney, and the lowest in the brain. In 3 livers and 10 kidneys, concentrations exceeded 3 mgTHg kg^−1^. In comparisons of THg and MeHg in the liver, kidney, muscle and brain, no significant differences were found between the age groups and between the birds coming from various voivodeships. In the whole, white-tailed eagle group medians of THg and MeHg concentrations were in descending order: K > L > M > B (Table 2). Unlike MeHg, differences were statistically confirmed between THg concentrations in the tissues (K–W test: *H* = 20.06, df = 3, *p* < 0.001) and concerned the following pairs of tissues: K–M, K–B and L–B. Although nephric THg concentration was almost twice as high as the liver, MeHg concentrations in these organs were very similar, as confirmed by the ratio MeHgK/MeHgL = 1. Respectively, in the brain and muscle, THg and MeHg concentrations were ~0.5 and ~0.7–0.8 of values recorded in the liver (Table 3). In all tissues of the white-tailed eagle, significant correlations (*p* < 0.001) between THg and MeHg concentrations were found (L: 0.721, M: 0.733, M: 0.926, B: 0.934).

**Table 2:** Concentration of total mercury (THg) and methylmercury (MeHg) in mg kg^−1^ dw in soft tissues of the white-tailed eagle *Haliaeetus albicilla* and osprey *Pandion haliaetus* from various voivodeships of Poland (*VZach* Zachodniopomorskie, *VWar-Maz* Warminsko-Mazurskie, *VLodz* Lodzkie, *im* immature, *subad* subadult, *ad* adult, *Med* median, *AM* arithmetic mean, *SD* standard deviation)

Origin of individuals and age group	Liver		Kidney		Muscle		Brain	
	THg	MeHg	THg	MeHg	THg	MeHg	THg	MeHg
*Haliaeetus albicilla*								
VZach: im								
*n*	4	4	4	4	4	4	4	3
Med	2.02	0.68	3.42	1.68	1.79	1.24	0.94	0.88
AM ± SD	2.00 ± 1.20	0.90 ± 0.84	3.26 ± 1.35	1.46 ± 0.94	1.93 ± 0.78	1.25 ± 0.62	1.04 ± 0.74	0.84 ± 0.53
Range	0.87–3.11	0.15–2.10	1.50–4.70	0.25–2.23	1.26–2.90	0.69–1.81	0.32–1.94	0.29–1.34
VZach: subad								
*n*	3	3	3	3	3	3	3	3
Med	2.01	2.01	3.36	0.96	0.97	0.64	0.56	0.34
AM ± SD	1.63 ± 0.79	1.63 ± 0.79	2.70 ± 1.50	0.81 ± 0.28	1.15 ± 0.46	0.67 ± 0.23	0.73 ± 0.32	0.43 ± 0.18
Range	0.72–2.16	0.72–2.16	0.99–3.76	0.49–0.99	0.81–1.68	0.45–0.91	0.54–1.10	0.32–0.64
VZach: ad								
*n*	8	8	8	8	8	8	5	4
Med	2.04	0.81	3.58	0.73	0.93	0.43	0.62	0.34
AM ± SD	1.96 ± 1.21	1.14 ± 1.08	4.33 ± 4.07	1.09 ± 1.06	1.41 ± 0.98	0.69 ± 0.50	0.96 ± 1.04	0.47 ± 0.44
Range	0.46–3.94	0.15–3.19	0.54–13.14	0.20–2.94	0.37–3.01	0.17–1.59	0.15–2.67	0.11–1.08
VZach: im + subad + ad								
*n*	15	15	15	15	15	15	12	10
Med	2.03	0.69	3.36	0.99	1.25	0.69	0.59	0.86
AM ± SD	1.91 ± 1.07	1.05 ± 0.90	3.72 ± 3.08	1.13 ± 0.90	1.50 ± 0.85	0.84 ± 0.53	0.93 ± 0.76	1.32 ± 1.19
Range	0.46–3.94	0.15–3.19	0.54–13.14	0.20–2.94	0.37–3.01	0.17–1.81	0.15–2.67	0.19–4.60
VWar-Maz: im								
* n* = 1	0.61	0.60	1.14	0.64	0.30	0.30	0.25	0.25
VWar-Maz: ad								
*n*	3	3	3	3	3	3	3	3
Med	0.62	0.37	1.11	0.34	0.45	0.45	0.41	0.21
AM ± SD	1.02 ± 0.88	0.75 ± 0.65	1.21 ± 0.92	0.43 ± 0.34	0.71 ± 0.54	0.45 ± 0.30	0.55 ± 0.43	0.31 ± 0.21
Range	0.40–2.03	0.37–1.50	0.34–2.18	0.15–0.81	0.36–1.33	0.15–0.75	0.21–1.03	0.17–0.56
VWar-Maz: im + ad								
*n*	4	4	4	4	4	4	4	4
Med	0.61	0.48	1.13	0.49	0.41	0.38	0.33	0.23
AM ± SD	0.92 ± 0.75	0.71 ± 0.54	1.19 ± 0.76	0.48 ± 0.30	0.61 ± 0.48	0.41 ± 0.26	0.47 ± 0.38	0.30 ± 0.18
Range	0.40–2.03	0.37–1.50	0.34–2.18	0.15–0.81	0.30–1.33	0.15–0.75	0.21–1.03	0.17–0.56
VLodz: ad								
*n*	3	3	3	3	3	3		
Med	1.30	1.25	3.55	1.01	1.19	0.35		
AM ± SD	3.76 ± 4.91	2.68 ± 3.09	17.7 ± 26.0	3.21 ± 4.19	2.91 ± 3.77	1.72 ± 2.43		
Range	0.57–9.41	0.57–6.23	1.81–47.6	0.30–7.23	0.30–7.23	0.29–4.53		
All individuals: VZach + VWar-Maz + VLodz								
*n*	22	22	22	22	22	22	16	14
Med	1.65	0.67	2.87	0.89	1.08	0.55	0.56	0.33
AM ± SD	1.98 ± 1.95	1.21 ± 1.36	5.16 ± 9.87	1.30 ± 1.70	1.53 ± 1.52	0.88 ± 0.95	0.82 ± 0.70	0.49 ± 0.37
Range	0.40–9.41	0.15–6.23	0.34–47.6	0.15–8.05	0.30–7.23	0.15–4.53	0.15–2.67	0.11–1.34
*Pandion haliaetus*								
VZach: im								
*n* = 1	3.65	2.33	4.03	3.25	2.00	1.71	0.33	0.32
VZach: ad								
*n* = 1	7.11	3.76	5.26	3.98	3.51	3.23	0.65	0.61
VZach: im + ad								
*n*	2	2	2	2	2	2	2	2
Med	5.38	3.04	4.64	3.62	2.75	2.47	0.49	0.46
AM ± SD	5.38 ± 2.45	3.04 ± 1.01	4.65 ± 0.87	3.62 ± 0.52	2.75 ± 1.07	2.47 ± 1.07	0.49 ± 0.23	0.47 ± 0.21
Range	3.65–7.11	2.33–3.76	4.03–5.26	3.25–3.98	2.00–3.51	1.71–3.23	0.33–0.65	0.32–0.61

**Table 3:** Indices concerning relations between THg and MeHg tissue concentrations in relation to THg and MeHg concentrations in liver in the white-tailed eagle *Haliaeetus albicilla* and osprey *Pandion haliaetus* found in Poland (*L* liver, *K* kidney, *M* muscle, *B* brain, *Med* median, *AM* arithmetic mean, *SD* standard deviation)

Species	THgK/THgL	MeHgK/MeHgL	THgM/THgL	MeHgM/MeHgL	THgB/THgL	MeHgB/MeHgL
*Haliaeetus albicilla*						
*n*	22	22	22	22	16	14
Med	1.77	1.00	0.78	0.70	0.51	0.49
AM ± SD	2.19 ± 1.44	1.13 ± 0.63	0.84 ± 0.34	1.09 ± 1.04	0.50 ± 0.17	0.71 ± 0.56
Range	0.75–5.66	0.41–3.43	0.37–1.62	0.19–4.60	0.26–0.86	0.36–2.06
*Pandion*-*haliaetus*						
*n*	2	2	2	2	2	2
Med	0.92	1.23	0.52	0.80	0.09	0.15
AM ± SD	0.92 ± 0.26	1.23 ± 0.24	0.52 ± 0.04	0.80 ± 0.09	0.09 ± 0.00	0.15 ± 0.02
Range	0.74–1.10	1.06–1.39	0.49–0.55	0.73–0.86	0.09–0.09	0.14–0.16

**Fig. 2:**
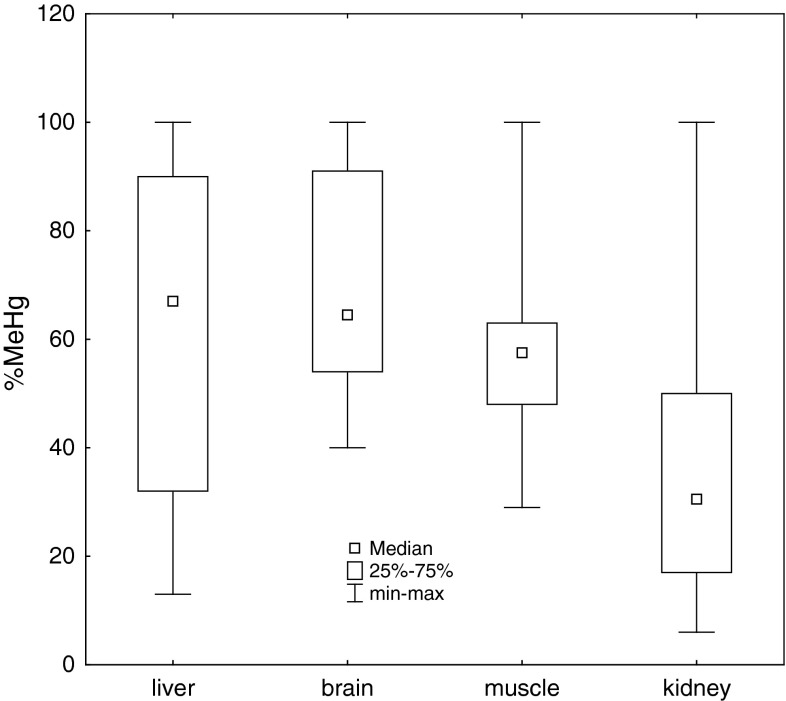
Percentage of MeHg in THg (%MeHg) in tissues of the white-tailed eagle found in Poland between 1996 and 2012

No differences in %MeHg related to age or place of origin of the white-tailed eagles were found but a certain trend for hepatic %MeHg was observed (K–W test: *H* = 6.0, df = 2, *p* < 0.06). The greatest %MeHg was detected in the liver of VLodz individuals and less in birds from VWar-Maz and VZach (96, 74, 55 %, respectively). The order of %MeHg medians in the tissues coming from all eagles was as following: L > B > M > K (Fig. 2) and the samples differed in %MeHg (K–W test: *H* = 16.8, df = 3, *p* < 0.001). In the kidney, %MeHg was significantly lower (30.5 %) than in the other tissues where medians were similar, from 51 % in the brain to 67 % in the liver.

Across the age groups, only the MeHgK/MeHgL index varied significantly (K–W test: *H* = 6.5, df = 2, *p* < 0.05). The values of this index among immatures, subadults and adults were as follows: 1.65, 0.75 and 0.94. The value in immatures proved to be much greater than adults, which was further confirmed by the M–W test (*U* = 8.0, *p* < 0.05). No statistical difference was observed between immatures and subadults due to low numbers of compared individuals (5 and 3, respectively).

### Osprey

In osprey, higher concentrations of THg and MeHg were found in samples coming from the adult than the immature (Table 2). The percentage of MeHg in THg in liver, kidney, muscle and brain was 58 % (range 53–64 %), 78 % (range 76–81%), 88 % (range 85–92 %) 95 % (range 94–97 %), respectively. Values of the other indicators are shown in Table 3. Only one of the indicators was >1 (MeHgK/MeHgL = 1.23), the others were <1.

## Discussion

In ecotoxicological reports, THg and MeHg concentrations are usually expressed per dry or wet weight, and therefore it is important to determine the water contents in the samples. However, such data are not often published for Falconiformes. Some authors assume 80 % moisture for all soft tissues or 70 % for the most frequently investigated liver (Hopkins et al. 2007; Rutkiewicz et al. 2011). Our study indicated that tissues of the white-tailed eagle do differ in water content, confirming the previous results of Falandysz et al. (1988) and Kalisinska et al. (2006). We propose, for Hg concentrations in Falconiformes, using soft tissues conversion rates that take into account differences in the tissue moisture (liver 70 %, kidney 75 %, muscle 70 %, brain 80 %).

In the EU, soft tissue samples of protected birds usually come from dead specimens sporadically found in the field (Kenntner et al. 2001; Lemarchand et al. 2012). Given the fact that Hg is incorporated into feathers only during their growth, and in falconiformes, feathers are shed gradually, and some as late as in the 3–4 years of life, Hg determined in feathers may come from a few weeks before or even dozens of months before, depending on the age of the individual and the stage of moulting (Johnels and Westermark 1969; DesGranges et al. 1998). Half-life of Hg in avian soft tissues is estimated to least 1–2 months, but depends on the amount and Hg species intaken with food—MeHg has a longer biological lifetime than InHg. That is why the liver and kidney are treated as important materials that reflect mid-term exposure to Hg (Hopkins et al. 2007; Scheuhammer et al. 2007).

Based on literature data (including white-tailed eagle), Shore et al. (2011) proposed indicative values of THg for the liver, kidney and brain of non-marine birds which may result in death at: >20, >40 and >15 (in adult) mg kg^−1^ ww (or >67, >160, and >75 mg kg^−1^ dw), respectively. Presently, such high lethal values of Hg concentrations in soft tissues (including the very seldom investigated brain) of the osprey, white-tailed eagle and its North American counterpart bald eagle are rarely recorded. In brains of those birds, THg concentrations even >10 mg kg^−1^ dw were observed sporadically both in Europe and North America, although in extremely rare case they reached 75 mg/kg (Jensen et al. 1972; Holt et al. 1979; Rutkiewicz et al. 2011). Unlike the brain, THg in the liver and kidney is frequently analysed. Hepatic and/or kidney investigations of European white-tailed eagles that found dead in the field curried out in the 1960–1980s (*n* = 85) and after 1990 (*n* = 102, including our results) revealed about 25 and 4 % of cases of mercury poisoning (Table 4). This comparison indicates a decrease in lethal Hg intoxication among white-tailed eagles in Europe over the last decades.

**Table 4:** Concentration of total mercury (mg kg^−1^ dw) in soft tissues of white-tailed eagle and osprey investigated in Europe (age categories: *nestl* nestling, *juv* juvenile, *im* immature, *ad* adult, *un* unknown, *n* range, *Med* median, *AM* arithmetic mean, *dl* detection limit)

Localisation and time	Age	Liver	Kidney	Muscle	Brain	References
White-tailed eagle *Haliaeetus albicilla*						
Finland, 1965		*n* = 5; 40–90	*n* = 5; 196–480	*n* = 5; 6.3–28.3		*Henriksson et al. (1966)
Germany, W, 1969	ad	*n* = 1; 161	*n* = 1; 106			*Koeman et al. (1972)
Germany, NE,	im-ad					*Oehme (1981)
1967–1976		*n* = 25; <dl-25	*n* = 23; <dl-57			
		Med 2.7	Med 23			
1976–1978		*n* = 10; 16–445	*n* = 10; 24–1224			
		Med 303	Med 462			
Sweden, 1965–1969	un			*n* = 7; 0.5–87	*n* = 5; 5.5–70	*Jensen et al. (1972)
Norway, 1972–1977	un	*n* = 24; 1.0–53	*n* = 24; 1.2–220	*n* = 24; 0.3–12		*Norheim and Frøslie (1978)
		Med 11	Med 14	Med 2.3		
Norway, 1965–1976	un	*n* = 25; <dl-53	*n* = 27; <dl-220	*n* = 27; <dl-13	*n* = 6; 1.0-10	*Holt et al. (1979)
		Med 8.0	Med 14	Med 2.3	Med 4.0	
Poland, 1982	un	*n* = 1; 30		*n* = 1; 2.8		Falandysz and Jakuczun (1986)
Poland, 1984	im	*n* = 1; 3.3	*n* = 1; 24	*n* = 1; 0.87		*Falandysz et al. (1987)
	ad	*n* = 1; 37	*n* = 1; 176	*n* = 1; 1.1		
Poland, 1986–1987	ad	*n* = 4; 2.9–110	*n* = 4; 10–224	*n* = 4; 1.6–21	*n* = 1; 4.2	*Falandysz et al. (1988)
Poland, 1987	ad			*n* = 1; 9.0		Dittmann et al. (1990)
Poland, 1991–1995	im-ad	*n* = 8; 0.6–21	*n* = 10; 1.4–220	*n* = 8; 0.02–5.6		Falandysz et al. (2001)
		AM 5.8	AM 52	AM 1.8		
Germany and Austria, 1993–2000	im-ad	*n* = 57; 0.03–12	*n* = 57; 0.3–144			*Kenntner et al. (2001)
		Med 1.2	Med 3.4			
Finland, 1994–2001	im-ad	*n* = 9; 4.6–30	*n* = 8; 11.3–211			*Krone et al. (2006)
		Med 10	Med 28			
Greenland, 1997–2000	juv-ad	*n* = 11; 1.9–19	*n* = 12; 2.6–14			*Krone et al. (2004)
		Med 4.7	Med 6.6			
Poland, 2005–2007	ad		*n* = 4; 0.4–4.3			Komosa et al. (2009)
			AM 1.5			
Poland, 2007	ad		*n* = 1; 3.5			Kitowski et al. (2012)
Poland, 1995–2012	im, ad	*n* = 22; 0.4–9.4	*n* = 22; 0.3–48	*n* = 22; 0.3–7.2	*n* = 16; 0.1–2.7	This study
		Med 1.6	Med 2.9	Med 1.1	Med 0.6	
Osprey *Pandion haliaetus*						
Norway, 1972–1977	un	*n* = 8; 2.7*–*60	*n* = 8; 9.6–180	*n* = 8; 1.7–9.3		*Norheim and Frøslie (1978)
		Med 15	Med 17	Med 4.7		
Norway, 1965–1976	un	*n* = 10; 2.0–60	*n* = 8; 2.4–180	*n* = 9; 1.3–9.3	*n* = 6; 1.0–8.5	*Holt et al. (1979)
		Med 9.3	Med 17	Med 3.3	Med 4.5	
Finland, 1970–1972	nestl	*n* = 2; 0.8–7.1	*n* = 3; 0.8–6.8	*n* = 3; 0.5–3.4	*n* = 3; 0.4–2.9	Häkkinen and Häsänen (1980)
France, 2007	juv-ad	*n* = 14; <dl-54				*Lemarchand et al. (2012)
		AM 11				
Poland, 2003–2006	im, ad	*n* = 2; 3.6–7.1	*n* = 2; 4.0–5.3	*n* = 2; 2.0–3.5	*n* = 2; 0.3–0.6	This study
		Med 5.4	Med 4.6	Med 2.7	Med 0.5	

* Original data in wet weight; in the recalculations on the dry weight was assumed 70, 75, 70, and 80 % moisture liver, kidney, muscle, and brain, respectively

Also Shore et al. (2011) proposed hepatic THg concentrations associated with adverse effects on avian reproduction (>2 mg kg^−1^ ww or >6.7 mg kg^−1^ dw). Only one of our 22 white-tailed eagles had a liver concentration >6.7 mgHg kg^−1^ (from VLodz) but 3 of 57 individuals collected in Austria and Germany (Kenntner et al. 2001). In our and Austro-German studies, the maximum values and medians of hepatic THg concentration were similar (9.4 vs. 12 and 1.6 vs. 1.2 mg kg^−1^ dw, respectively; Table 4). Among southern Baltic countries, the largest number of white-tailed eagles nest in eastern Germany and northern Poland (Hauff and Mizera 2006). That is why studies in border areas in Poland and Germany have given most data on Hg in this species. In Kenntner et al. (2001), 43 of 57 (75 %) specimens came from eastern Germany (Fig. 1). Eagles in the border area range freely across tens of kilometres (Hauff and Mizera 2006; Table 1). Moreover, in eastern Germany and Poland, Hg-pesticides were banned at a similar time (Kenntner et al. 2001). For these reasons, the results of mentioned authors and our findings show many similarities. In the white-tailed eagles from Germany and Poland, higher THg concentrations were found in the dried kidney than the liver (Germany: 3.4 vs. 1.2 mg kg^−1^, Poland: 2.9 vs. 1.6 mg kg^−1^, respectively). In 26 German eagles, THg residues were ~3.3 ppm in the liver, kidney, or both, but only one had THgK/THgL ≤ 1. Thus, 46 % of the birds had a value of this index ≥1 (Kenntner et al. 2001). In the Polish group of white-tailed eagles, 10/22 individuals (45 %) had an index >1. In the German and Polish eagles with lower THg concentrations (<3.3 ppm) in the organs, such strong differences were not found between hepatic and nephric THg concentrations. However, those concentrations significantly correlated with each other in both studies.

In comparison to the German and Polish studies, white-tailed eagles in Finland and Greenland (collected between 1994 and 2001) had THg concentrations 5 and 2 times higher in the liver, and 9 and 2 times higher in the kidney (Table 4). Such large differences between the Polish-German and Finnish populations could have resulted from three main factors in the Finnish population: age (domination of older individuals), the site (Baltic coast and islands) and diet composition and origin, mostly marine fish and aquatic birds associated with Hg-contaminated water bodies (Krone et al. 2006). In the case of the Greenland population, the increased accumulation of Hg in the white-tailed eagles may have resulted from their diet of marine origin (seabirds and fish) with a naturally high content of Hg (Krone et al. 2004). Compared to the Finnish and Greenland populations, eagles living in the Poland and Germany have a more inland character and inhabit mainly south Baltic areas rich in lakes and forests, and minimally contaminated with Hg. Eagles in these areas benefit from a more varied diet, including fish, waterfowl, and carrion (Kenntner et al. 2001).

In Europe, data on Hg in osprey’s soft tissues are extremely scarce. In four papers were investigated 35 ospreys in total. Both before 1980 and after 2000, in single ospreys examined in Norway and France, high hepatic THg concentrations (50–60 mg kg^−1^ dw) were reported, which could have contributed to their death (Table 4).

In some papers on piscivorous Falconiformes, relations between the age and tissue Hg concentration were found. For example, positive correlations were revealed between age and kidney or brain THg concentrations in the white-tailed eagle and bald eagle, respectively (Krone et al. 2006; Rutkiewicz et al. 2011). Moreover, Krone et al. (2004) showed a greater concentration of nephric THg, but not hepatic THg, in adult white-tailed eagles than the younger, and suggested that the higher Hg level in the kidney than the liver was caused by a long-term accumulation in the kidney, mainly as InHg. In the liver of ospreys from France, Lemarchand et al. (2012) revealed a significant increase of THg concentrations between non-flying juveniles and subadults or adults, and a significant decrease between subadult and adult groups. A study on osprey from the U.S. showed age-dependent relationships between THg concentrations in the liver and kidney. Generally, adults had higher and more variable THg residues than chicks and hatch-year ospreys (Hopkins et al. 2007). In our white-tailed eagles, correlations between age and the Hg tissue concentrations were not significant. This discrepancy in the results is likely to depend on the intensity of the transfer of MeHg to feathers during their growth (especially in chicks), the level of accumulated Hg and the efficiency of MeHg demethylation. Ratios between MeHg and InHg change dynamically in avian soft tissues, and are conditioned by the amount of MeHg intake and absorption in the intestine, tissue concentrations, and the ability to demethylate and excrete Hg. Especially, in fish-eating birds, there are significant interspecific and interindividual differences in the intensity of MeHg demethylation (Gochfeld 2003; Scheuhammer et al. 2008). Therefore, information on hepatic and nephric THg concentrations is not sufficient for diagnosing MeHg toxicity in birds (Scheuhammer et al. 2007; Rutkiewicz et al. 2011). Kidney THg concentrations are known to represent mainly InHg, and THgK/THgL ≥2 proves such exposure (Scheuhammer et al. 1998; Gochfeld 2003). However, if nephric and hepatic THg concentrations are low, as in the majority of birds examined in this study, then THgK/THgL may be <2. In our eagle group, THgK/THgL = 1.8 and MeHgK/MeHgL = 1 but %MeHg was over two times higher in the liver than kidney (67 vs. 30 %). In the muscle and brain, %MeHg levels were similar (~60 %) and both significantly differed from nephric level (Fig. 2). Norheim and Frøslie (1978), based on kidney THg residues, divided white-tailed eagles from Norway into two groups: I (*n* = 7), and II (*n* = 5). Nephric concentration in the group I was below 33 mgHg kg^−1^ dw and in group II was above that level. The hepatic and nephric medians in the group I were similar (~7 mgHg kg^−1^) but in muscle much lower (1.3 mgHg kg^−1^). Index %MeHg/THg in the tissues was in the range 48–59% with the lowest and highest values in the liver and muscle. In the group II, THg concentrations in all tissues were higher (16, 88, 3.7 mg kg^−1^) but the lowest and highest values of %MeHg had the kidney (7%) and muscle (65%), intermediate the liver (37%). Although the Norwegian eagles from groups I and II hepatic and nephric THg concentrations were 4.5/10 and 2.5/30 times greater than in analogous tissues of the Polish samples, differences in %MeHg were more ambiguous. The lowest hepatic %MeHg (37%) was observed in eagles from group II (with high median: 16 mgTHg kg^−1^) and Polish individuals (~48 and 67%) with lower medians (7.3 and 1.6 mgTHg kg^−1^, respectively). In bald eagles from North America, hepatic mean THg concentrations ranged from 7 to 14 mg kg^−1^ dw (Weech et al. 2003; Rutkiewicz et al. 2011), alike in white-tailed eagles from group I investigated in Norway, and much greater than the hepatic THg concentration in our eagles (<2 mg kg^−1^). The mean fraction of MeHg in the liver of the bald eagle and white-tailed eagle from the group I was similar (50–60%), but in the white-tailed eagles from 1996 to 2012, it was higher (67%). The cited and compared above values of THg and %MeHg in both eagle species confirm observations by various authors who report that the increasing concentration of THg in avian liver is accompanied by a decrease in %MeHg in this organ. However, negative correlations between hepatic THg and %MeHg have not always been observed (Scheuhammer et al. 1998; Weech et al. 2003).

Comparing the species in analogous age categories, in the eagle species, lower Hg concentrations in the liver and/or kidney were generally found than in the osprey investigated in Europe and North America (Norheim and Frøslie 1978; Weech et al. 2003; Scheuhammer et al. 2007; Hopkins et al. 2007; Lemarchand et al. 2012). It may partly arise from differences in their diets. Both white-tailed eagle and bald eagle are opportunistic feeders but osprey is obligatory fish-eating predator, so is more susceptible to the accumulation of Hg.

Out of 13 ospreys collected in France and Poland in 2000s, seven (54 %) had a hepatic THg concentration associated with an adverse effect on avian reproduction. Hepatic THg concentrations in subadult and adult groups from France and Norway were similar (~15.7 mg kg^−1^) although the ospreys came from 2000s and 1970s (Table 4). It seems that in Europe Hg exposure of osprey has not decreased.

The share of hepatic and nephric MeHg in waterbirds varies within wide ranges and depends on environmental factors and the age/size/physiological condition of individuals, the species and trophic group (DesGranges et al. 1998; Scheuhammer et al. 2007). In contrast to the liver and kidneys, it is believed that in avian muscle, MeHg demethylation process occurs to a small extent, and therefore the MeHg share is very high (>80%). This was confirmed among piscivorous ospreys, mergansers and loons (Norheim and Frøslie 1978; Scheuhammer et al. 1998; Hopkins et al. 2007; Kalisinska et al. 2014). In contrast to these species, the mentioned Norwegian groups I and II of the white-tailed eagle and the Polish one, despite some differences in THg concentrations in muscle (1.3, 3.7 and 1.1 mg kg^−1^, respectively), had muscle %MeHg similar (~60%) and much lower than for previously studied piscivorous birds. Our and Norwegian investigations indicate the existence of a relatively intense demethylation of MeHg in the tissue of white-taile eagle, and document a greater interspecific variability that previously thought. In our study, values of %MeHg were similar in the eagle’s muscle and brain (~60%). Although the brain THg level was low (0.6 mg kg^−1^), the %MeHg was the same as in the bald eagle from the U.S. Great Lakes, despite its higher THg level (2.8 mg kg^−1^ dw) (Rutkiewicz et al. 2011). In the bald eagle, it was demonstrated that demethylation of MeHg increased as THg in the brain increased, like in the liver (Scheuhammer et al. 2008; Rutkiewicz et al. 2011).

Scientists agree that MeHg easily crosses the brain–blood barrier and strongly affects a brain and the neuromuscular synapses of warm-blooded vertebrates (Clarkson and Magos 2006; Scheuhammer et al. 2007, 2008). Experimental studies on the impact of small amounts of Hg in the avian brain, carried out at biochemical and behavioural levels, have shown that MeHg concentrations in the range of even 0.2–1.0 mg kg^−1^ dw in the fish can lead to disorders in individuals and have ecological consequences. Eating such a diet probably does not always result in moderate or large Hg concentrations in avian brains and other tissues. However, even low Hg concentrations in brain can cause alteration of pairing behaviour, slowing down the escape response (for example, from an oncoming train), too late recognition of dangers during flight, such as overhead power lines and wind power rotors at the growing number of wind farms in the EU (Krone et al. 2006; Frederick and Jayasena 2011).

## Conclusion

This study helps to broaden knowledge on the degree of Hg intoxication in two rare bird species in Europe. In the past 25 years, there has been a decline in THg concentrations in soft tissues of white-tailed eagles among Baltic populations, especially Polish**–**German ones. It has been shown that among the piscivorous birds, there is considerable variation in the share of MeHg in THg. Moreover, %MeHg in the muscle of the white-tailed eagle is significantly lower (~60%) than in other piscivorous birds (>80%). Unlike the white-tailed eagle, it seems that in the European osprey concentrations of THg in soft tissues have not decreased over the period, but comparative data for this species are extremely scarce.
